# Integrating Genomic and Phenomic Approaches to Support Plant Genetic Resources Conservation and Use

**DOI:** 10.3390/plants10112260

**Published:** 2021-10-22

**Authors:** Gayle M. Volk, Patrick F. Byrne, Clarice J. Coyne, Sherry Flint-Garcia, Patrick A. Reeves, Chris Richards

**Affiliations:** 1 United States Department of Agriculture, Agricultural Research Service, National Laboratory for Genetic Resources Preservation, Fort Collins, CO 80521, USA; Pat.Reeves@usda.gov (P.A.R.); Chris.Richards@usda.gov (C.R.); 2 Department of Soil and Crop Sciences, Colorado State University, Fort Collins, CO 80523, USA; patrick.byrne@colostate.edu; 3 United States Department of Agriculture, Agricultural Research Service, Western Regional Plant Introduction Station, Pullman, WA 99164, USA; Clarice.Coyne@usda.gov; 4 Plant Genetics Research Unit, United States Department of Agriculture, Agricultural Research Service, Columbia, MO 65211, USA; Sherry.Flint-Garcia@usda.gov

**Keywords:** breeding, database, genebank, genotype, phenotype

## Abstract

Plant genebanks provide genetic resources for breeding and research programs worldwide. These programs benefit from having access to high-quality, standardized phenotypic and genotypic data. Technological advances have made it possible to collect phenomic and genomic data for genebank collections, which, with the appropriate analytical tools, can directly inform breeding programs. We discuss the importance of considering genebank accession homogeneity and heterogeneity in data collection and documentation. Citing specific examples, we describe how well-documented genomic and phenomic data have met or could meet the needs of plant genetic resource managers and users. We explore future opportunities that may emerge from improved documentation and data integration among plant genetic resource information systems.

## 1. Introduction

**Figure 1:**
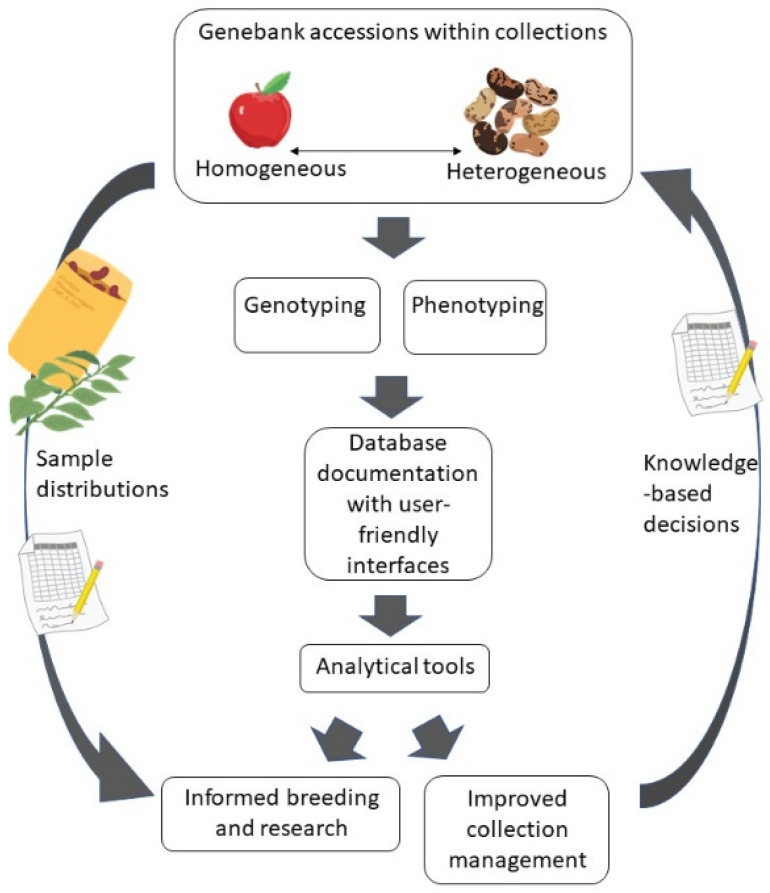
Diagram of selected genebank processes. Genebank accessions range from homogeneous varieties to heterogeneous seedlots. Genomic and phenomic data are collected, documented and analyzed. With this information, improved collection management is possible and specific samples can be requested for breeding and research.

Genebanks offer a broad range of plant genetic diversity for use in research and breeding programs. For decades, crop researchers have collected phenotypic trait data on genebank accessions. New high-throughput technologies facilitate the collection of phenomic data (large-scale, often multi-dimensional, phenotypic datasets). Similarly, smaller-scale DNA marker data have been eclipsed by more comprehensive genomic data for characterizing collection genetic diversity. With these large datasets, the challenges of digital information management are becoming as important as managing the physical collection of germplasm. Databases that integrate data types, promote standardized data collection and documentation methods, incorporate appropriate analytical tools and provide user-friendly access will help curators and users of plant genetic resources (PGR) to manage, locate and identify diversity of agronomic and horticultural importance (Figure 1). Tanksley and McCouch [1] and, more recently, the Divseek Initiative advocate for more effective use of genebank diversity; however, the genetic resources stored in genebanks are still underutilized [2,3]. This largely stems from the challenge of identifying useful accessions in large and diverse ex situ collections.

Genebank accessions are genetically complex, with a range of genetic profiles. Homozygous/homogeneous seed samples include inbred lines and accessions derived by single seed descent. These include elite cultivars (e.g., wheat [*Triticum aestivum* L.] variety ‘Jagger’ and maize [*Zea mays* L.] inbred line ‘B73′). An example of homozygous/heterogeneous accessions are landraces of self-pollinating crops that are comprised of an assortment of different genotypes. Heterozygous/homogeneous accessions include clonally maintained but originally outcrossing crops (e.g., ‘Granny Smith’ apple [*Malus domestica* Borkh.]). Heterozygous/heterogeneous accessions are represented by wild species accessions and outcrossing landraces. As wild species accessions are regenerated, the extent of heterozygosity may decrease [4]. An understanding of the relative level of within-accession genetic variation is important to successfully regenerate genebank accessions, collect and document phenotypic and genotypic data, and use genebank materials in breeding and research programs.

## 2. Customer and Stakeholder Needs for Plant Genetic Resources

Plant breeders, geneticists, biologists, and educators have different levels of genetic knowledge and access to different kinds of field and laboratory facilities and software tools. Nevertheless, there is an overall need for a wide range of PGR, especially novel germplasm (i.e., materials with genetic variation that are not readily available in existing breeding populations or cultivars) that is viable, disease-free, and has acceptable legal conditions for use [5,6]. To access these resources, an easily navigated search and request system is necessary. Characterization and evaluation data based on standardized methods and rating systems help narrow down the search to materials that meet objectives.

Improved web interfaces that provide genebank inventory identities, along with genotype and phenotype data, can guide accession selection. Many users of genebank materials would find it helpful to know the allelic states of major genes (or associated molecular markers) that control important crop-specific traits (e.g., *Rht*, *Ppd*, and *Vrn* genes for plant stature and flowering time in wheat [7]). Plant breeders would be aided by the availability of traits from wild or unadapted germplasm introgressed into an adapted genetic background or introduced “exotic” germplasm that has been adapted to particular locations via recurrent selection and crosses with adapted germplasm. Examples include products of pre-breeding/genetic enhancement programs such as the Germplasm Enhancement of Maize project (see description below) or panels of synthetic hexaploids in wheat [8].

DNA sequence data for accessions [9], as well as a reference genome for the crop of interest (e.g., [10,11]), are increasingly available. Whole-genome estimates of genetic diversity and population structure of germplasm panels (e.g., [12]), and genome-wide association study (GWAS) results for relevant traits and germplasm (e.g., [13]), can generate data for identifying the accessions best suited for particular purposes. In addition, detailed phenotypic evaluation data for both grower-oriented and consumer-targeted traits (e.g., unpersoned aerial vehicle (UAV) data of accessions under heat or drought stress; in-depth data on health-promoting properties) will have a more limited number of users, but at least a subset of customers would be able to leverage these types of datasets. Plant breeders will benefit from genomics-assisted breeding software to facilitate the introgression of desired genomic regions into breeding material and for genomic selection [5,14].

## 3. Genomic and Phenomic Approaches Help Meet Customer and Stakeholder Needs for Plant Genetic Resources

While germplasm collections are extraordinarily valuable for geneticists and breeders, there are several challenges for efficient use of these collections. For example, it is often unclear how accessions are related genetically or how adapted they are to a given environment. Many accessions were received decades ago, sometimes with scant or contradictory passport data (e.g., pedigree, provenance, and place of origin). Modern genomic and phenomic tools can be applied to collections to guide germplasm choice for target traits and environments.

### 3.1. Genomic Tools for Elucidating Germplasm Relationships

Genomic tools such as high-density single-nucleotide polymorphism (SNP) genotyping and whole-genome resequencing can describe the diversity in collections and reveal relationships among accessions. Nearly 4400 samples representing approximately 2500 inbred maize lines known as the “Ames Panel” in the USDA collection were genotyped with 680,000 SNPs (using genotyping by sequencing; GBS) and evaluated for a core set of traits in a field trial at three locations in 2010 [15,16]. Analyses revealed the population structure within the collection: popcorn and sweet corn accessions formed distinct subpopulations separate from the remaining temperate germplasm, as did the tropical germplasm [16]. There was also a clear pattern based on geographic origin. Within U.S. germplasm, genetic differentiation occurred from north to south consistent with adaptation to day length. Tropical lines contained the highest level of diversity in the collection, consistent with previous reports. These data also described the diversity of various subsets, relative to the whole collection. For example, the 282-inbred line Goodman association panel [17] captured 75% of the diversity of the whole collection, and the founders of the nested association mapping population [18] captured 57% of the diversity in the whole collection, attesting to their value in exploring maize inbred diversity. Inbred lines from US and Canadian public breeding programs represented 83% of the diversity in the collection, while private germplasm with expired Plant Variety Protection (PVP) certificates contained only 45% of the diversity in the collection, reflecting a focus on maintaining the three main heterotic patterns in temperate maize. The success of these endeavors was due in part to the homogeneous nature of the inbred lines that were assessed. Future progress for other crops is dependent upon having tools available for heterogeneous accession types.

#### 3.1.1. Germplasm Enhancement of Maize (GEM) Program for Pre-Breeding

It is difficult to introgress unadapted accessions into desirable backgrounds for target environments. Evaluating all accessions for a crop across multiple environments can require vast resources and time. The Germplasm Enhancement of Maize (GEM) program is a collaboration between private industry, USDA and university partners that aims to increase the diversity of US maize germplasm [19]. Fifty-one maize landrace accessions with agronomic merit and high yield potential [20] were the starting materials for the GEM Program beginning in 1995. In the GEM traditional protocol, an exotic accession is crossed with an elite corn belt dent inbred line from a private cooperator, marked only as belonging to the stiff stalk or non-stiff stalk heterotic group. The F1 is then crossed to a second inbred line in the same heterotic group by either the same or different cooperator. The resulting progeny are self-pollinated for several generations and undergo evaluation/selection each season to eliminate disease or insect susceptibility. A moderate number of selected S2 families are testcrossed to the opposite heterotic group to make hybrids for yield trials in a small number of locations. The best 10 families (selection intensity ~3%) are self-pollinated and testcrossed with numerous testers for yield trials in a larger number of locations. The GEM Program typically releases approximately 10 GEM lines per year, which are used primarily for corn breeding by private companies. This program continues to be strongly supported by small and large multi-national corn breeding companies, demonstrating its value to the industry. This approach for pre-breeding could be extended to other crops where there is a need for a broader genetic base and where public and private sectors agree to expend resources for the common good of the crop community.

In addition to the traditional GEM breeding protocol, the Allelic Diversity project was started in 2005 to create a resource for gene discovery, allele mining, and genomic research beginning with ~600 maize landraces, regardless of *a priori* agronomic merit. Each landrace accession was crossed and backcrossed into both a stiff stalk (PHB47) and non-stiff stalk (PHZ51) background. Inbred lines were then produced by either doubled haploid methods [21] or self-pollination. To date, approximately 500 inbreds have been released to the public as a new genomic resource. The Allelic Diversity inbreds have been used to conduct GWAS for flowering time and plant height [22], root system architecture [23], and kernel composition traits where novel loci have been identified [24] reflecting the wide diversity of this unique resource.

#### 3.1.2. Subsets for Allele Mining

The origin of the core subset concept traces its roots to the 1970s and 1980s when genebank holdings were continuing to increase from institutional exchanges and active collecting missions for landrace and wild material [25,26]. The influx of new and diverse accessions was vital for filling gaps in global collections, but the rise in holdings had the unintended effect of making management decisions relating to monitoring and regeneration more difficult. It also made the selection process more complicated for users, who had to navigate thousands or tens of thousands of accessions with little more than a species designation and basic passport information.

Breeding programs make use of collection subsets. For example, the Ames Panel of maize was used for GWAS of numerous traits [27,28,29,30]. Subsets of the Ames Panel are often created based on adaptation to a specific environment; the Wisconsin Diversity Panel consists of lines that flower and mature within the shorter growing season of the northern United States [31]. The Wisconsin Diversity Panel has been used for several GWAS experiments [32,33,34,35,36,37]. Prior to the availability of genomic data, subsetting the Ames Panel would have required either phenotypic datasets from common garden experiments or relied on curator knowledge of the entire collection.

It has become easier to collect genomic data for genebank collections than to perform phenotypic evaluations. For genebank accessions that are genetically homogeneous, it is acceptable to sample only a single or several individuals for whole-genome genotyping. In contrast to the maize example, genotyping one or several individuals for a heterogeneous accession is of limited utility because (1) their multi-locus genotypes cannot be retrieved because all individuals are different, (2) small samples provide poor estimates of allele frequencies, and (3) rare alleles will not be discovered for all loci.

**Figure 2:**
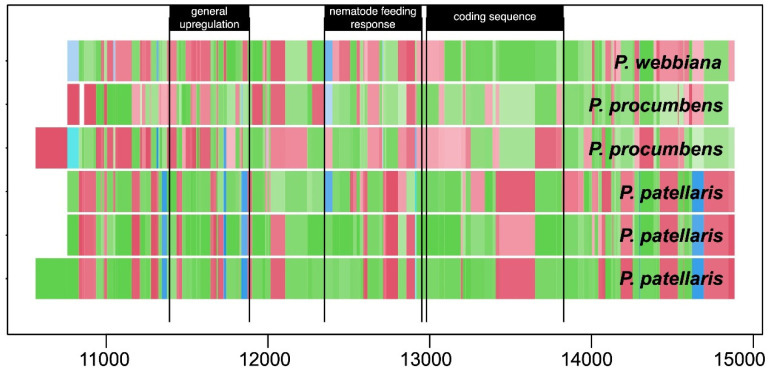
Allele mining in the tertiary gene pool of sugar beet [*Beta vulgaris* L.]. The cyst nematode resistance gene Hs1*^pro−1^* from *Patellifolia procumbens* (C. Sm.) A.J. Scott, Ford-Lloyd & J.T. Williams. is conveyed to sugar beet in a large translocation. A promoter sequence activated by nematode feeding makes this locus a target for engineering the disease response in sugar beet [40]. Major allele frequency differences across the genomic region containing Hs1*^pro−1^*, recovered from the pooled sequencing pan-genomes of six wild *Patellifolia* spp. populations, are shown. Colors indicate different allelic variants, shading within a color indicates variant frequency within the pool (lower = lighter). Substantial variation in the nematode responsive region can be mined from these populations. Whole-genome pooled sequencing data from 202 cultivars, breeding lines, wild relatives, and genebank accessions of *Beta vulgaris* is available under NCBI BioProject PRJNA563463.

For cataloging genetic variation in heterogeneous accessions, whole-genome pooled sequencing offers a cost-effective alternative to independent sequencing of multiple individuals (e.g., [38]; Figure 2). Pooled sequencing captures whole-genome sequence diversity segregating among the individuals of an accession. The resulting pan-genome-like data structure can be interrogated using genomic position or sequence homology (e.g., BLAST). With adequate sampling (~100 individuals per pool) and adequate sequencing depth (~1x per individual), pooled sequencing allows estimation of allele frequencies genome-wide with an accuracy equal to or better than sequencing individuals, at a lower cost [39]. As collection-wide genotyping projects commence [3], pooled sequencing data can be accumulated one accession at a time for all accessions of a species, with each completed dataset improving insight into the genetic structure of a collection. Allele frequencies, or probabilities of recovering haplotypic variants, are delivered to the genebank customer in the form of heterogeneous germplasm. Providing the means for users to query allele frequencies at any locus of interest in advance of requesting germplasm would be of enormous utility.

Modern crop improvement programs augment traditional crosses and field evaluations with transgenic techniques and gene editing. To enable these approaches, comprehensive sequence data for genebank accessions is important. Whole-genome pooled sequencing datasets support this use by providing information on the full complement of sequences available for individuals in an accession, ensuring gene editing targets are present and avoiding off-target effects [41]. Pooled sequencing data that have been processed into genome-wide SNP or short haplotype frequencies describe accession diversity more practically for breeding programs and correlate logically with phenotypic characterization and evaluation data held in genebank databases, which are usually measured at the population, not individual, level [42].

#### 3.1.3. Gene Discovery—GWAS Using Genebank Collections

Genome-wide association analysis has enabled PGR to contribute more extensively to marker/gene discovery in most species. Diversity subsets from genebank collections are a readily available resource to perform GWAS using a range of markers from simple sequence repeats (SSRs) to SNPs from GBS or SNP arrays [15]. Whole-genome sequencing (WGS) of diversity panels is underway (e.g., rice [*Oryza sativa* L.] [43]; soybean [*Glycine max* (L.) Merr.] [44]; chickpea [*Cicer arietinum* L.] [45]) and perhaps in the near future WGS will be available for an entire crop collection. Statistical approaches and their software implementations are improving [46]. GWAS is becoming possible for any variable trait that can be precisely phenotyped in genebank accessions, thus increasing the value of genebank collections.

### 3.2. Phenomics in Applied Breeding

Plant breeders and researchers require an understanding of the phenotypic/phenomic data that are available. This includes documentation about how the data were collected, such as the numbers of individuals sampled and experimental field designs (particularly for heterogeneous accessions), as well as the use of standardized descriptors and ontologies. Most of the traits important to breeders exhibit significant genotype × environment interactions so the full environmental context under which phenotypic measurements were made is necessary [47,48]. High-throughput phenotyping (HTPP) in plant breeding programs (e.g., [49]) provides a model for HTPP of large numbers of PGR accessions across multiple environments by genebanks and cooperators in the future [50]. Efforts are underway to improve data presentation and availability, an example is the AgBioData project, which will harmonize data storage and interoperability across databases [51].

Beginning in the 1990s, an evolution occurred from simple, formal phenotypic descriptors to the machine-readable ontologies available today, wherein the controlled vocabulary includes not only trait definitions but also relationship terms, so that complex phenotypic information can be processed by computer. Phenotypic descriptors for PGR have been published for over 100 crops under the auspices of the CGIAR centers IPGRI and Bioversity International (now The Alliance of Bioversity International and CIAT). Bioversity used these to develop crop ontologies and the Crop Ontology Curation Tool [52]. These ontologies are not available for all crops, such as pea, *Pisum sativum* L., and many fruits. However, pea and other new crop ontologies are under development. Other harmonious crop ontologies can be found on Planteome [53] and AgroPortal [54]. The goals of all these efforts are data interoperability to assist in meeting FAIR (findability, accessibility, interoperability, and reusability) data standards [55]. As genebanks collect data using internationally recognized standard formats, data become more accessible and specific accessions can be selected for use in breeding programs. This also facilitates accession comparisons among different genebanks.

## 4. Genebank Curation Needs

High-quality genebank collections contain well-curated passport, phenotypic, and genotypic data. Acquiring these data is challenging due to resource limitations and the vast size of most genebank collections. Success depends upon partnerships between genebanks and user communities. As they become available, genomic and phenomic data can help guide collection management and improve the value of the collection. These data permit the identification of collection gaps, which can be filled with new acquisitions [56]. They also can help curators maintain the genetic integrity of accessions. Knowledge of within-accession variation helps define optimal regeneration and storage strategies to minimize the effects of genetic drift [4].

Genomic and phenomic data can provide knowledge applicable to curation, such as whether accessions are correctly assigned to taxon, whether they are redundant, or whether they differ when they should not [56]. They can help quantify changes accumulated during ex situ conservation or changes occurring in situ, in the wild, since the accession was collected. Genomic and phenomic data can be used to ensure that cultivar identities are consistent with names used in other genebanks and user communities. At times, curators and their advisory groups must make difficult management decisions because resources are insufficient to accommodate ever-expanding collections. Genomic and phenomic data can help guide decisions to ensure that the purpose and goals of genebank collections are met, even if some accessions must be eliminated. Finally, genomic and phenomic data can help guide users in selecting the best genebank materials for their purposes.

## 5. Genomic and Phenomic Approaches Help Meet Curation Needs

### 5.1. Genomic Data Improve Collection Management

Availability of accession-level genomic data for genebank collections has facilitated taxonomic identification of unusual or hybrid species. Lentils, which include *Lens* species and *Lens culinaris* Medik. sub-species, are difficult for non-taxonomists to identify. Wong et al. [57] used sequence data from GBS to correct species and sub-species identifications and identify interspecific hybrids of wild accessions of lentil.

Genotyping the maize Ames Panel revealed several interesting findings related to curation [16]. First, to assess intra-accession variability, approximately 2200 duplicate inbreds (as determined by accession name) were genotyped. Of these, 98% were determined to be at least 0.99 identical by state (IBS), the threshold used to determine that two accessions were “identical”. Redundant accessions could be removed in order to simplify curation efforts. Numerous accessions were determined to be isogenic or nearly isogenic (>0.97 IBS). For example, 50 inbreds had IBD > 0.97 with B73, a historically important inbred line. Genebank managers and stakeholders can weigh costs and benefits when determining which collections of near isogenic lines should be maintained.

Genomic data have provided useful information about genetic gaps in collections. Previously, geographic coverage was the primary criterion for targeting plant acquisitions and assembling core collections. Correlation of geographic gap-filling and genomic gap-filling has shown the advantage of using genomic data. The IPK genebank analyzed GBS data for 21,405 barley accessions to identify gaps in the collection. They found “a pronounced under-representation of some regions of the world in IPK’s wild barley (*Hordeum vulgare* subsp. *spontaneum* C. Koch.) collection” [58]. Duplicates were also identified, most likely from merger of former East and West German collections. The International Potato Center genebank genotyped 250 potato (*Solanum tuberosum* L.) accessions with a 12K SNP array and identified putative misclassified accessions [59]. GBS data for 441 lettuce accessions showed that crisp head lettuce (*Lactuca sativa* L.) PGR in the USDA collection lack genetic diversity, hindering genetic advances in this important crop [60].

### 5.2. Phenomic Data Improve Collection Use

**Figure 3:**
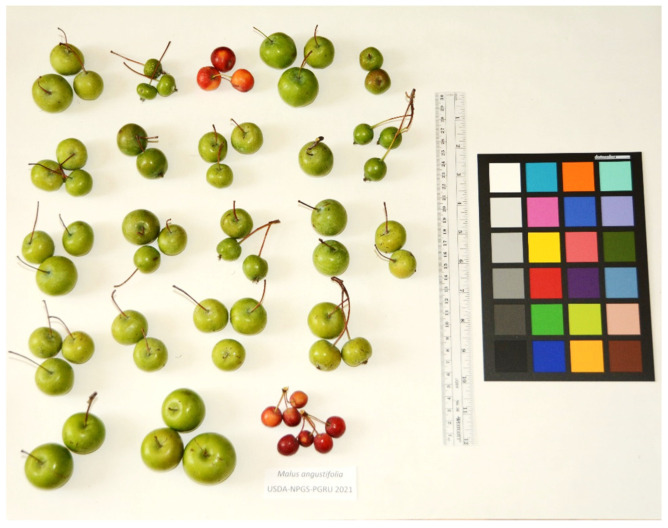
Montage of fruit of *Malus angustifolia* (Aiton) Michx. accessions in the USDA-NPGS apple collection in Geneva, NY. The red fruits are not characteristic of the species. Taxon assignment will be confirmed using genotyping by sequencing [63].

Access to phenotypic/phenomic data allows users to select specific materials based on traits of interest, such as resistance to specific diseases, resistance to abiotic stress, or quality components. Replicated, multi-year trials using standardized methods are preferred [61], although often a single data point for a single year may be all that is available. Uploaded images of fruits, seeds or other parts of interest, with scale bars and color standards, provide valuable information to determine accession heterogeneity, trueness-to-type (at the accession or species level; Figure 3), and value to breeding programs [62]. Comparison of images can determine if accession phenotypes are consistent with original submission descriptions or other historical records.

The traits addressed by HTPP technologies are broad, from well-established disease assessment [64,65], to tree and plant architecture [66,67], to protein content in wheat [49]. Satellite images of wheat [68], UAVs [69], tractor mounted (e.g., [70]) and handheld detectors (e.g., [71,72]), and between-row ultracompact robots [73] comprise a set of new technologies potentially valuable for HTPP of genebank accessions.

Although genebank HTPP is in early stages, examples of completed studies are emerging that efficiently provide needed evaluation data to the user community. A diversity panel of lentil was phenotyped in seven countries for three years, providing insight into photothermal interactions to guide future production expansions [74]. HTPP deployed in the Canadian lentil trials (six environments) contributed to a data depth that was not possible in the non-HTPP sites [71]. This type of data analysis could be adapted for other genebank tasks such as seed germination scans. The International Potato Center deployed a UAV remote sensing and multi-spectral camera to accurately predict maturity and productivity of potatoes [75]. In the future, UAV and robots could collect field data for large collections at multiple timepoints in the growing season [76].

Several examples are available for HTPP of seeds. A true HTPP seed imaging platform with a conveyor belt and barcode reader was developed to record the color, size and shape dimensions of the small-seeded lentil [77]. A similar approach for soybean lacks the third dimension and automation aspects [78]. A mobile application was developed for grain width and length HTPP in the field [79]. Maize ear/kernel HTPP has two published platforms [80,81]. Commercial software was used to scan pulse crop PGR seed images, but throughput is limited as seed must be manually separated [82]. SmartGrain software developed for rice eliminates this impediment by excluding overlapping grains and removes awns and pedicels [83].

## 6. Data Integration for Plant Genetic Resource Curation and Use

Genebanks have implemented inventory management databases that associate accessions with passport, image, phenotypic and genotypic data. Resource limitations have resulted in phenotypic and genotypic data that are not comprehensive within or across crops. There are also a number of challenges in associating and retrieving heterogeneous accessions with their corresponding phenotypic/phenomic and genotypic/genomic data within databases. These challenges must be overcome to ensure well-documented, standardized data are available for improving genebank management and utility.

To efficiently exploit the variation contained in genebank collections, there have been efforts to develop sophisticated information management systems. These systems primarily provide database, analytical and decision support capabilities to genomics driven breeding projects. Capabilities include managing and tracking physical and digital descriptor data and, increasingly, integrating genomic sequence data, field observations of phenotypic data, including digital image and spectral data. Platforms include the Genomic Open-source Breeding Informatics Initiative [84], Breeding Insight [85], Excellence in Breeding [86], and Breedbase [87,88,89]. A common API (application programming interface) defines protocols for interoperability among breeding applications and databases (BrAPI, [90]). Informatics projects focused on defined sets of crops such as Seeds of Discovery [91,92] and Agent [93] provide important data integration among genomic and phenotypic projects. These projects also promote collaborative networks to connect genebank collection information with populations from breeding and pre-breeding projects. Software platforms such as Germinate [94] and the IPK’s Bridge web portal [95] focus on data visualization and integrating gene bank genomic and phenotypic data.

Crop-specific or clade-oriented genomic databases (e.g., MaizeGDB) offer numerous visualizations and capabilities connected to genebank accessions. Maize SNP data were used to establish an IBS relationship matrix among 2800 inbred lines, and this matrix was used to populate a tool called “TYPSimSelector” [96,97], where the user can query for inbred lines that are most closely related or most diverged from the inbred line of interest. Users can search for a replacement for an accession because of a defect in a trait, such as disease or insect susceptibility.

Looking forward, data curation will likely involve the use of persistent identifiers (PIDs) such as Plant Introduction (PI) numbers or Digital Object Identifiers (DOI) for sample identification. Through the adoption of BrAPI, the GRIN-Global information management system can become interoperable with Breeding Insight and crop-specific databases. Several databases, including Bridge IPK [98] and Germinate [99], have already successfully merged some components of genebank and breeding information management.

## 7. Future Prospects

Current and future genebank users, as well as curation teams, will increasingly require access to high-quality genomic and phenomic data. Access to genetic diversity information could help ensure phenotypic data are collected from an adequate number of individuals. Integration of genomic and phenomic data for heterogeneous accessions requires special attention to DNA polymorphism and elevated or complex patterns of phenotypic variance. Technological advances in information management systems have focused primarily on specific crops. Future efforts should consider the relative heterogeneity of genebank accessions and how it can be effectively managed when data are collected. Systems must be user-friendly and widely applicable to diverse customers. In addition, they must be adaptable and scalable, to support new genomic and phenomic technologies. Future research should focus on ensuring that tools are available to effectively use genomic and phenomic data for informed curation decisions.

Increasingly sophisticated artificial intelligence methods and sequence data lead to the question of whether every accession must be phenotyped to choose accessions for gene(s) or trait(s) of interest. Advances in statistical prediction may change how characterization data can select germplasm for further evaluation. FIGS (Focused Identification of Germplasm Strategy) applies machine learning (ML) algorithms and environmental data to identify candidate accessions associated with a trait of interest [100]. In Bari et al. [100], non-phenotyped wheat accessions carrying rust resistance were predicted based on the trait-environment relationship in other accessions. Similarly, ML approaches can be used to assemble germplasm subsets that maximize variation of haplotype blocks associated with phenotypic variation [101,102,103]. Genomic prediction is an increasingly useful tool in specialty crops such as pea [104] and well-resourced collections such as barley [105]. A training population with both genotypic and phenotypic values is the basis for developing a statistical model. The model then predicts the phenotypic values of lines using only genotypic data. A genomic selection application in sorghum (*Sorghum bicolor* (L.) Moench) demonstrated the power of this approach for selecting accessions for high biomass from a large PGR collection [64]. Improvement of genomic prediction algorithms and increasing genomic data coverage across PGR accessions will advance their use by genebank managers and genebank users alike [106,107].
